# Application of the Gaussian Mixture Model to Classify Stages of Electrical Tree Growth in Epoxy Resin

**DOI:** 10.3390/s21072562

**Published:** 2021-04-06

**Authors:** Abdullahi Abubakar Mas’ud, Arunachalam Sundaram, Jorge Alfredo Ardila-Rey, Roger Schurch, Firdaus Muhammad-Sukki, Nurul Aini Bani

**Affiliations:** 1 Department of Electrical and Electronic Engineering Technology, Jubail Industrial College, Al Jubail 35718, Saudi Arabia; sundaram_a@jic.edu.sa; 2 Prince Saud bin Thunayan Research Centre, Royal Commission for Jubail, Al Jubail 35718, Saudi Arabia; 3 Department of Electrical Engineering, Universidad Técnica Federico Santa María, Santiago de Chile 8940000, Chile; jorge.ardila@usm.cl (J.A.A.-R.); roger.schurch@usm.cl (R.S.); 4 School of Engineering & the Built Environment, Edinburgh Napier University, Edinburgh EH10 5DT, Scotland, UK; f.muhammadsukki@napier.ac.uk; 5 Razak Faculty of Technology and Informatics, Universiti Teknologi Malaysia, Jalan Sultan Yahya Petra, Kuala Lumpur 54100, Malaysia; nurulaini.kl@utm.my

**Keywords:** gaussian mixture models, electrical trees, partial discharge

## Abstract

In high-voltage (HV) insulation, electrical trees are an important degradation phenomenon strongly linked to partial discharge (PD) activity. Their initiation and development have attracted the attention of the research community and better understanding and characterization of the phenomenon are needed. They are very damaging and develop through the insulation material forming a discharge conduction path. Therefore, it is important to adequately measure and characterize tree growth before it can lead to complete failure of the system. In this paper, the Gaussian mixture model (GMM) has been applied to cluster and classify the different growth stages of electrical trees in epoxy resin insulation. First, tree growth experiments were conducted, and PD data captured from the initial to breakdown stage of the tree growth in epoxy resin insulation. Second, the GMM was applied to categorize the different electrical tree stages into clusters. The results show that PD dynamics vary with different stress voltages and tree growth stages. The electrical tree patterns with shorter breakdown times had identical clusters throughout the degradation stages. The breakdown time can be a key factor in determining the degradation levels of PD patterns emanating from trees in epoxy resin. This is important in order to determine the severity of electrical treeing degradation, and, therefore, to perform efficient asset management. The novelty of the work presented in this paper is that for the first time the GMM has been applied for electrical tree growth classification and the optimal values for the hyperparameters, i.e., the number of clusters and the appropriate covariance structure, have been determined for the different electrical tree clusters.

## 1. Introduction

Electrical treeing is a key degradation phenomenon of high-voltage polymeric insulation [1]. When electrical trees are initiated, they grow until they bridge the entire insulation material, resulting in catastrophic failure of the power system plant. Electrical trees are strongly related to partial discharge (PD) activity, which is usually characterized using techniques such as phase-resolved PD (PRPD) patterns [2,3], and, to a lesser extent, pulse sequence analysis (PSA) [4], pulse waveform analysis [5], and nonlinear time series analysis [6,7].

When PD activity is severe, there is higher dissipation of energy and greater PD amplitudes, resulting in tree growth, and serious degradation [8,9]. As mentioned in the literature [10], PD activity might be undetectable when the tree structure is forming conductive channels within the insulation system, therefore, thorough knowledge of PD behavior related to treeing degradation is needed, especially for conditioning monitoring engineers in the industry. Understanding the tree phenomenon is crucial in determining the remaining lifetime of an electrical asset.

Several studies analyze tree growth from PD activity [10,11,12,13,14]. In particular, Lv et al. [10], Bao et al. [11], Zhou et al. [14], and Alapati [13] investigated PD development during the early stages of PD degradation in epoxy resin insulation, cross-linked polyethylene (XLPE) cable, and low-density polyethylene (LDPE). These studies found that the growth rate of electric trees is strongly influenced by the increasing level of voltages. However, for electrical tree propagation in an XLPE cable, the skewness (i.e., the extent to which the distribution deviates from the normal distribution) of the maximum amplitude-phase distributions decreased with the spread of the electrical stress, and the skewness can be considered as a parameter to fully identify different levels of electrical tree propagation. Furthermore, the tree pattern feature in cross-linked polyethylene (XLPE) cable is similar to the needle-plate electrode system.

In the case of the LDPE, there was a considerable decrease in PD repetition rate and PD magnitude of LDPE filled with alumina nanocomposites compared to unfilled LDPE. There was an increase in the PD inception voltage with 3% weight of the filler loadings and then decreases when the filler loadings reached 5%. On the other hand, other researchers investigated tree propagation mechanisms in XLPE cable insulation based on a double electrical tree structure [12]. It was found that five types of electrical tree structures (branch, forest, bine-branch, pine-branch, and mixed configurations) will propagate in XLPE cable insulation due to the effect of the irregular congregating state, differences in the crystalline structure, and the presence of residual stress in the semi-crystalline polymer.

Few investigations are found in the area of pattern identification of PD characteristics from the initial stage to breakdown of the insulation due to electrical treeing. Park et al. [15] evaluated and classified PD degradation of electric trees for cable insulation. The authors of this study utilized three classification techniques: Adaptive neuro-fuzzy combination (ANFIS), multi-layer perceptron (MLP), and principal component analysis (PCA). Compared to other defects, such as voids and metal surfaces, the findings specifically demonstrated different features of electric trees. The results clearly showed distinct characteristics of electrical trees as compared to that of other defects such as voids and metal surfaces. Among all the classification techniques, ANFIS showed higher identification potential and can be used for classifying electrical tree progression with about a 99% recognition rate. In another investigation, Salama et al. [16] applied a MLP neural network (NN) to discriminate between PD defects in voids and electrical trees. In particular, the algorithm could recognize discharge patterns from different degradation levels of the electrical trees. Although this algorithm was applied for the case of real power cable faults, it has not been applied to electrical tree faults degradation up to the breakdown stage. In another study, Park et al. [17] attempted to recognize three different electrical tree models using the adaptive network-based fuzzy inference system. The models considered samples with a needle-plane electrode, needle-void-plane electrode, and needle-metal strip-plane electrode. Statistical features extracted from the aforementioned tree models were applied as inputs to the ANFIS system. The results showed a good discrimination rate of these models up to 100%. However, this work was limited to electrical tree patterns only, without analyzing the progression and different stages of tree growth.

This paper studies the growth of electric trees in samples of epoxy resin under various voltage levels aiming to correlate PD activity with the stage of tree growth through the analysis of PRPD patterns using a Gaussian mixture-based model (GMM) clustering technique. GMM was chosen over other techniques because it is flexible and can perform hard clustering for complex data. Using this approach, it is expected to assess the remaining life of insulation subjected to electrical treeing degradation more accurately.

Section 2 details the experimental setup and the data capture procedure, describing the dataset, and their analysis. Section 3 explains the GMM model used in this work. Section 4 is the data processing technique adopted, while Section 5 describes this work’s results and conclusion.

## 2. Experiment and Data Capture Procedure

### 2.1. Experimental Setup

**Figure 1:**
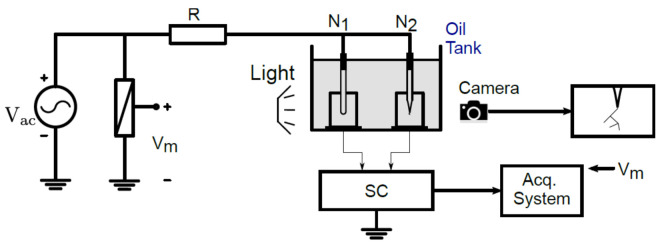
Test circuit for electrical tree growth experiments.

The samples were prepared using the conventional needle-to-plane configuration with a gap distance of ~2 mm between the needle tip and the bottom of the sample. The needle was a hypodermic needle Terumo with an approximate curvature radius of 3 µm, the insulating material was epoxy resin (Mepox–1685/L, a Bisphenol A diglycidyl ether (epoxy system) in Santiago, Chile and the cuboid dimensions were 10 × 10 mm base and 25 mm height. Electrical treeing experiments were carried out using the test circuit shown in Figure 1. The voltage source (Vac) was a transformer fed from the grid through a variac (Variable AC Transformer). The samples were fed through a limiting resistance (R) in order to reduce disturbances and protect the instruments in case of breakdown. PD measurements were carried out using the balanced circuit shown in standard IEC 60,270 [18]. The treeing sample (N2) and the dummy sample (N1, PD free) were placed into a transparent oil container to prevent unwanted surface discharges and allow visualization of tree growth using an optical camera. The signals from the treeing and dummy samples were subtracted in the subtracting circuit (SC), whose output was fed to a commercial PD system (Acquisition System) that continuously registered PD activity. The voltage was measured using a voltage divider (Vm), which was also used by the PD measurement system. The minimum value of PD magnitude for the measurement was set to 2 pC; however, to reduce the background noise recorded, a threshold between 10 to 15 pC was used for the analysis.

Before the electrical tree growth experiment itself, an incipient electrical tree needed to be created in each sample. To initiate an electrical tree, 12–16 kV 50 Hz voltage was applied to each sample until the camera optically observed a tree, and then the voltage was turned off and the sample was prepared for the tree growth experiment. By doing this, the initiation stage was separated from the propagation stage, which was the stage to be analyzed in this research. The electrical tree growth experiment was carried out in the selected samples by applying 12, 14, and 16 kVrms 50 Hz until breakdown, according to Table 1, where also the resulting time-to-breakdown (Time BD) is shown. This time is the duration of tree growth from the initial stage until final breakdown of the insulation. PD measurements were made using two simultaneous means: continuous recording with filming camera and taking pictures every 10 s. The utilization of this simultaneous registry system was required to correlate PD behavior (electrical response of the insulation) and tree propagation shape/length (physical damage).

### 2.2. Partial Discharge Recorded and Selected Data for Analysis

**Figure 2:**
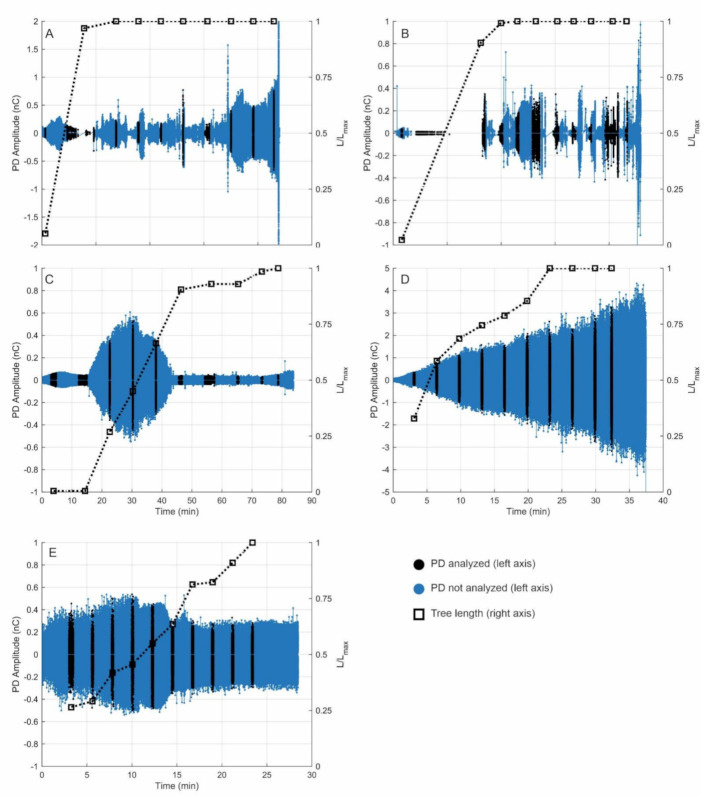
Time series of partial discharge (PD) amplitude and tree length for each sample: (**a**) Sample A, (**b**) Sample B, (**c**) Sample C, (**d**) Sample D, and (**e**) Sample E different.

The measurements of the electrical tree growth experiment are shown in Figure 2, where the PD amplitude time series (left axis) and the tree length progression (right axis) are compressed in the same graph for each sample. Tree length was extracted from the tree images taken during the growth and was measured as the furthest tree extent from the needle tip in the direction of the plane electrode. In the graph, the length was represented in per unit values, i.e., the ratio between the length (L) and the length of the first tree branch that reached the plane electrode (L_max_). Note that dielectric breakdown did not occur immediately after the tree arrived at the counter electrode. In particular, in the cases of Samples A and B, a considerable amount of experimental time passed after the tree bridged the insulation.

Although PD was recorded during the entire experiment, tree growth analysis was carried out using ten selected windows or intervals of analysis to study the parameters’ evolution during tree growth. The selection of data and intervals followed the criteria previously described by Zheng et al. [19]. Each interval was selected to have at least 10,000 PD events and at least 10 s of continuous measuring time. In practice, this resulted in a total of 10,000–60,000 PD events (observations) per analysis interval for all the samples. The first analysis interval was chosen to start three minutes after the beginning of the test for a more stable PD activity, and the last interval was set to finish at least five minutes before the breakdown. The separation between intervals depended on the duration of each test. The intervals of analysis are shown as black bands in Figure 2a–e.

**Figure 3:**
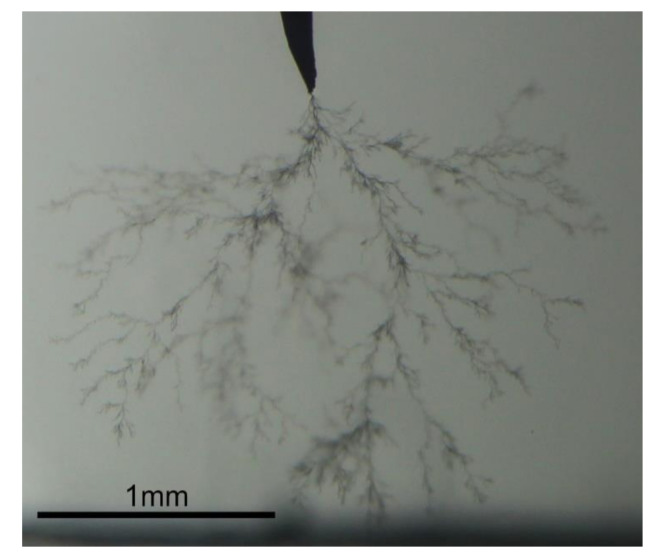
Picture of Sample B at interval 2, showing a filamentary tree growth.

The results indicate that PD dynamics are different for every sample, depending on the stressing voltages and the stage of tree growth. For example, Samples A and B had irregular trends, and Sample B even had periods of no detected PD while the tree was growing. This phenomenon has been reported before and is due to the growth of ‘filamentary’ trees [19]. This is observed in Figure 3; though Samples B and C were both stressed at 14 kV, time series of PD amplitudes had different behavior, which was also observed when comparing Samples D and E, stressed at 16 kV. In particular, Sample D showed the highest PD amplitude values among all the samples, with a constantly increasing trend.

**Figure 4:**
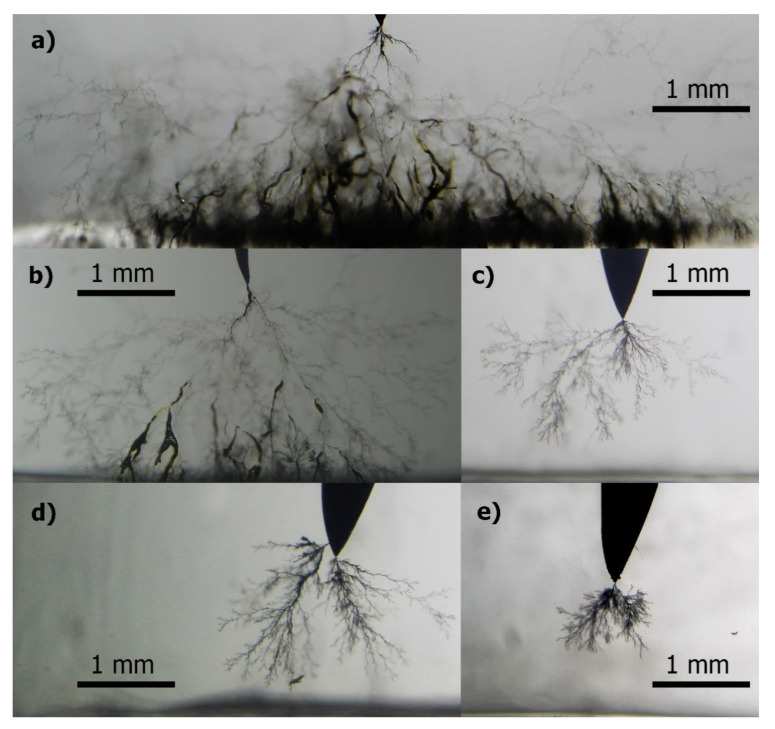
Images of electrical trees at interval 6 in each sample: (**a**) Sample A, (**b**) Sample B, (**c**) Sample C, (**d**) Sample D, and (**e**) Sample E.

Images of the electrical trees of each sample at interval 6 are presented in Figure 4. It can be observed how Sample A, aged at 12 kV, presented the widest electrical tree. It is worth noting that the images are from the same interval (6th), but they do not correspond necessarily to similar stressing time; for Sample A, the 6th interval was at 130 min of aging, which is longer than any other total stressing time.

## 3. Gaussian Mixture Model Clustering Technique and Classification Model

Clustering techniques have been widely used in power system analysis and Rajabi et al. [20] have discussed a literature survey of various clustering techniques available and their application towards smart metering. Out of all the available models for unsupervised learning, the most popular is k-means clustering, which groups data according to a distance-based calculation with respect to a centroid [21]. The centroids are updated iteratively through a mean value and the clustered data will be in a circular shape. The drawback of the k-means clustering is that it fails to cluster data that are not in a circular shape, such as elliptical shape or irregular patterns. This drawback is overcome by GMM, which uses a probability density function (PDF) determining parameters by expectation-maximization (EM) technique. Compared to the k-means, the centroid formed by GMM takes into account the mean as well as the variance of the data, accommodating different sized clusters with varying correlations within them [22].

The clustering of unimodal distribution and multimodal distribution using GMM is explained in [22,23,24]. The comparison in [25] reveals that GMM takes more simulation time than k-means. Additionally, GMM can group complex patterns into similar components that match closely while k-means uses simple principles to produce only abstract information. The performance and comparison of the sampling methods used in GMM are reported in [26].

GMM can also be used for both hard and soft clustering of the dataset. In hard clustering, the GMM assigns each query data point to a particular cluster, which will maximize the posterior probability of the component given the data. In soft clustering, the GMM calculates the likelihood of the query data point belonging to a specific cluster and then assigns the query data point to a cluster, which would have maximum posterior probability, calculated using Bayes’ theorem. In this study, the versatile soft clustering GMM is utilized for unsupervised learning to model unknown data distribution by multivariate normal distributions. Unfortunately, k-means clustering has no means to measure the likelihood or uncertainty of cluster assignments. On the other hand, GMM uses probability distribution functions that can model any input dataset by assigning each point a probability to belong to a certain cluster. Hence, it is used for clustering in this work.

The various steps in the GMM are explained in [22]. The Gaussian model is formulated by Equations (1) and (2). Let $X\;=\;\left\{x_{1},x_{2},\cdots x_{n}\right\}\;$be a set of n observations. The variable $x_{i}\;$is distributed among a mixture of M components. The PDF of $x_{i}$ is written as shown in Equation (1), which is the weighted sum of Gaussian densities given by Equation (2) and the sum of weights $\sum_{i\;=\;1}^{M}w_{i}\;=\;1,$ $w_{i}\;$represents the mixing probabilities.

(1) $$p\left(x_{i}|\lambda \right)\;=\;\overset{M}{\underset{i\;=\;1}{\sum }}w_{i}g\left(x_{i}|\mu_{i},{\;\Sigma }_{i}\right)$$

(2) $$g\left(x|\mu_{i},{\;\Sigma }_{i}\right)\;=\;\frac{1}{{\left(2\pi \right)}^{D/2}{\left|\Sigma_{i}\right|}^{1/2}}exp\left\{-\frac{1}{2}{\left(x\;-\;\mu_{i}\right)}^{T}\Sigma_{i}^{-1}\left(x\;-\;\mu_{i}\right)\right\}$$

where M is the number of Gaussian densities, x-D is the dimensional continuously valued data vector, *w_i_*, I = 1…M is the weight of the mixture,$g\left(x|\mu_{i},{\;\Sigma }_{i}\right),i\;=\;1,2\ldots M\;$is the component of Gaussian densities, μ is the mean vector of dimension D, Σ is the covariance matrix of dimension D×D, and $\lambda_{i\;}=\;\left\{w_{i},\mu_{i},{\;\Sigma }_{i}\right\}\;\mathrm{is}\;\mathrm{the}$ parameter of the GMM. In this study of clustering for insulation degradation using GMM the following covariance structure is adopted:

1. The covariance structure of the components will determine the shape and orientation of the ellipsoid drawn over the cluster. The covariance matrix is diagonal instead of being full to avoid the over-fitting problem, and major and minor axes of the ellipsoid are parallel and perpendicular to the abscissa and the ordinate. The covariance matrix is shared among the components; hence, the ellipse of each cluster has the same size and orientation.
2. The expectation-maximization (EM) algorithm fits the GMMs. The initial values of the parameters are set, and then the initial cluster assignments for data points are allowed to be selected randomly.
3. Regularization is applied in order to avoid the likelihood of data point becoming ill-conditioned and starts moving towards infinity.

### 3.1. The Expectation Maximization (EM) Algorithm

Expectation maximization (EM) is a mathematical algorithm used to find the correct parameters for a model. The estimated parameter of mean, variance, and weight are necessary to cluster the data, but this is possible only if the Gaussian family is known. The EM algorithm starts with random parameters, and then the optimal parameters are found by iteration. This algorithm has the capability to deal with latent variables. Assuming *k* clusters are to be assigned, then *k* distributions are required with mean and covariance values of µ1, µ2, …, µk and Σ1, Σ2, …, Σk, respectively. The EM algorithm generally has two main steps, i.e., the Expectation step (E-step) and the Maximization step (M-step) [27].

#### 3.1.1. The Expectation Step (E-step)

In this step, using randomly initialized parameters, for every point x_j_, we obtain the likelihood of belonging to a certain cluster c_1_, c_2_, ..., c*_k_*. This is achieved using Equation (3).

(3) $$r_{jc}=\;\frac{probability\;that\;x_{j}belongs\;to\;c}{sum\;of\;probability\;x_{j}\;belongs\;to\;c_{1},c_{2}\ldots c_{k}}\;=\;\frac{\pi_{c}\;N\left(x_{j}\;;\;\mu_{c}\;,\Sigma_{c}\right)}{\Sigma_{c^{\prime }}\pi_{c^{\prime }}N\left(x_{j}\;;\;\mu_{c^{\prime }}\;,\Sigma_{c^{\prime }}\right)}$$

This value would be high if the point is allocated to the correct class, or vice versa.

#### 3.1.2. The Maximization Step (M-step)

In this step, the parameter λ is updated as follows:

1. The weight is updated using Equation (4), which is the ratio of cluster points to the overall number of points.
  (4) $$w_{c}\;=\;\frac{Number\;of\;points\;assigned\;to\;a\;cluster}{Total\;numberof\;points}$$
2. Then, the covariance and the mean values are modified using Equations (5) and (6) in relation to the probability values for the data point and based on the values assigned to that particular distribution. Therefore, any data point having a high probability of being a member of the distribution should be contributing a higher portion.
  (5) $$\mu_{c}=\frac{1}{Number\;of\;points\;assigned\;to\;cluster}\Sigma_{j}r_{jc}x_{j}$$
  (6) $$\Sigma_{c}=\frac{1}{Number\;of\;points\;assigned\;to\;cluster}\Sigma_{j}r_{jc}{\left(x_{j}-\mu_{c}\right)}^{T}(x_{j\;}-\mu_{c})$$

Based on the updated parameters the E-step is repeated. These two steps are iterated until the optimal parameters are obtained using the log-likelihood function as described in [3].

## 4. Data Processing

Data pre-processing is an important step in machine learning models. To explain the data pre-processing steps, dataset A at the first interval (A1) is used. Sample A1 analysis is summarized in Table 2. The dataset has 10,139 observations with minimum values of Phi (phase angle) and Q (PD amplitude) are −1.790 × 10^−5^ and −7.220 × 10^−11^, respectively. The maximum values of Phi and Q are 9.997 × 10^−1^ and 8.880 × 10^−11^, respectively. In the data pre-processing step, the data is transformed using the normalized function available in MATLAB, which transforms the data with a mean of 0 and standard deviation of 1. The transformed variables Phi (Stdscale) and Q (Stdscale) are used by the clustering algorithm, which has a mean of 0 and standard deviation of 1 as shown in Table 2.

## 5. Results and Discussion

### 5.1. Gaussian Mixture Model Clustering

GMM clustering was applied to generate clusters for every training pattern from the initial stage to the breakdown of the insulation, for each dataset. The overall aim was to be able to understand and classify different PD patterns during the stages of tree growth. The important hyperparameters in GMM are the number of clusters *k* and appropriate covariance structure Σ. In GMM the covariance structure includes a covariance matrix, which can be diagonal or full, and the nature of the covariance matrix, which can be shared or unshared. When a diagonal covariance matrix is chosen, the minor and major axes of the confidence ellipsoids drawn over the clusters are parallel or perpendicular to the x and y axes. When a full covariance matrix is chosen, there is no restriction to the orientation of the minor and major axis of the confidence ellipsoids drawn over the clusters. A shared covariance matrix indicates that all confidence ellipsoids have the same size and orientation, whereas an unshared covariance matrix indicates different sizes and shapes of the confidence ellipsoids. Choosing the appropriate hyperparameter is a very important task, and the method adopted is presented in the next section.

#### 5.1.1. Hyperparameter Tuning of GMM

**Figure 5:**
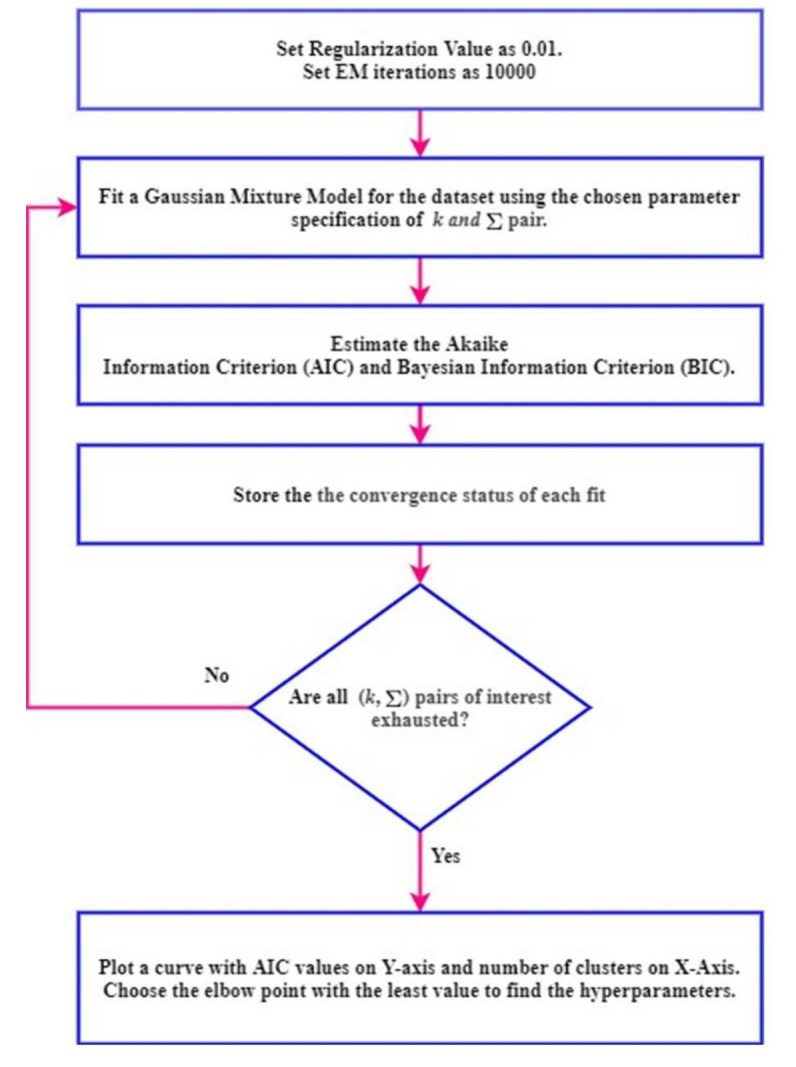
Procedure to choose the hyperparameters.

The number of cluster components *k* and appropriate covariance structure Σ is unknown for each stage of electrical treeing in Samples A−E. The most commonly used technique to tune the hyperparameters is by comparing the Akaike information criterion (AIC) and Bayesian information criterion (BIC). AIC is the relative distance between the unknown true likelihood function of the data and the fitted likelihood function of the model. A lower AIC means the model is closer to reality. BIC is an estimate function of the posterior probability of a model being true under certain assumptions, so a lower BIC means the model is bound to be the genuine model. A detailed discussion on the importance of AIC and BIC related to model selection is available in [28]. The procedure to choose the optimal values for the hyperparameters namely the number of clusters *k* and appropriate covariance structure Σ is shown in Figure 5 and was coded using MATLAB. A regularization value of 0.01 is specified and the EM iteration is specified as 10,000 in order to avoid ill-conditioning of the covariance matrix during EM iteration. The number of clusters *k* takes a value from 1 to 12.

**Figure 6:**
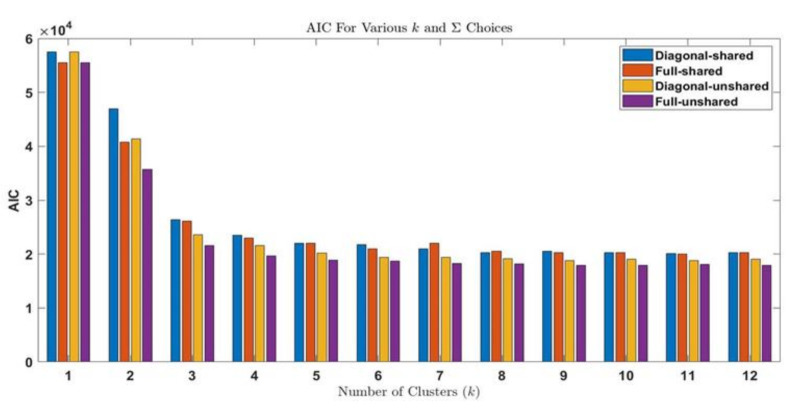
Bar Plot of Akaike information criterion values for each fit in dataset A1.

**Figure 7:**
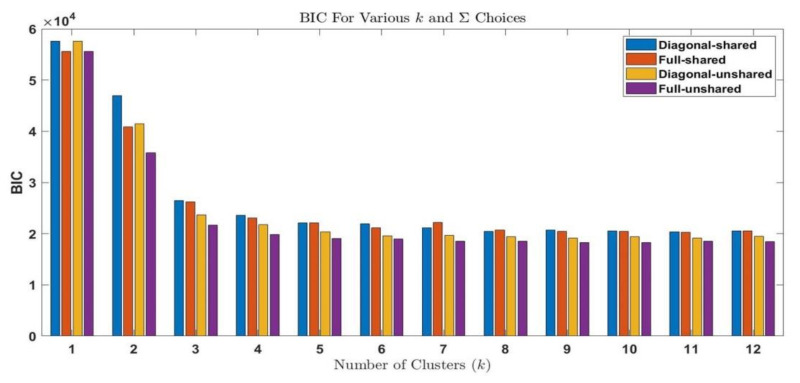
Bar Plot of Bayesian information criterion values for each fit in dataset A1.

**Figure 8:**
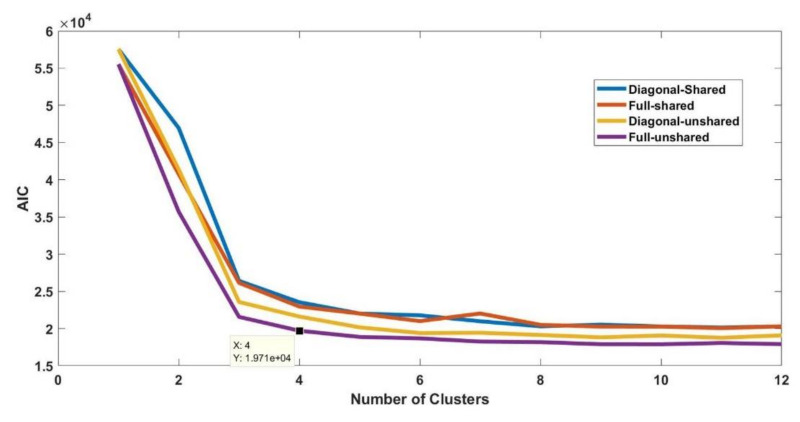
Plot of Akaike information criterion values for each fit in dataset A1.

**Figure 9:**
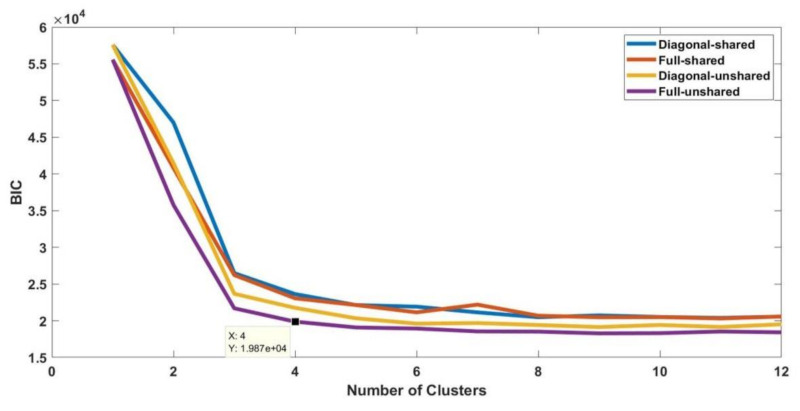
Plot of Bayesian information criterion values for each fit in dataset A1.

The procedure shown in Figure 5 is performed for all fifty datasets. The procedure is explained for the initial stage of electrical treeing in Sample A in the first interval and the same procedure is applicable for other datasets. For sample A1, the AIC and BIC values are summarized in Table 3 and Table 4, respectively. The bar plots grouped by the number of clusters are shown in Figure 6 and Figure 7, respectively. From the bar plot it is very clear that when the number of clusters is more than 1, the AIC and BIC values decrease, and after a specific number of clusters is reached, the variation of AIC values is significantly low. The point where the change in AIC value declines the most is the elbow point. The elbow point in Figure 8 and Figure 9 was determined by plotting a curb, and corresponds to the number of clusters *k* = 4 and covariance structure which is full and unshared. These are the best hyperparameters for dataset A1. The same procedure has to be repeated in all datasets and the best hyperparameter for each dataset in samples A to E are provided in Table 5, Table 6, Table 7, Table 8 and Table 9, respectively.

According to Table 5, Table 6, Table 7, Table 8 and Table 9, the minimum value of *k* is 4 and its maximum value is 8. The nature of the covariance matrix is mostly full with an unshared structure and rarely diagonal with an unshared structure. The values of hyperparameters selected for the GMM are shown in Table 10.

The shared covariance is false, which indicates a non-identical or unshared covariance matrix. The grid length is important in order to draw the confidence ellipsoids over the clusters. Grid length and the number of iterations for the EM algorithm are selected by trial and error.

### 5.2. GMM Results and Discussions

**Figure 10:**
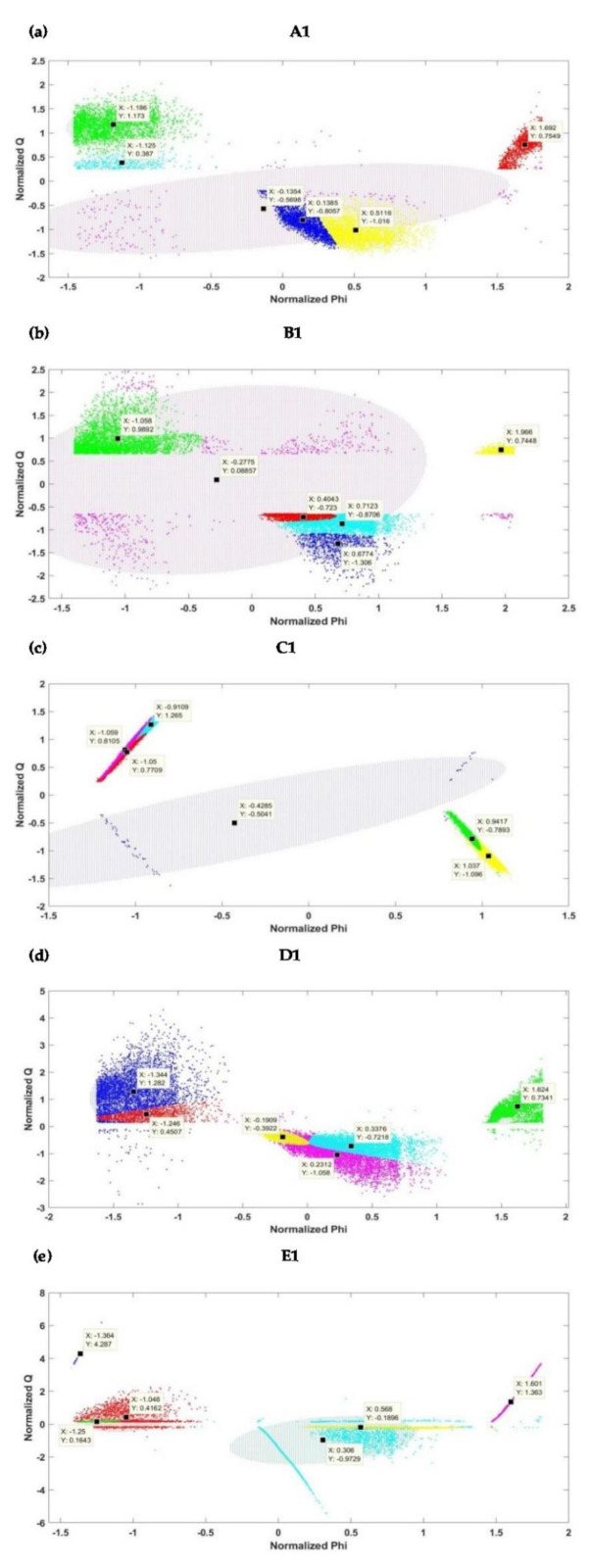
Clusters for the different datasets at the initial stage of degradation (**a**) Sample A, (**b**) Sample B, (**c**) Sample C, (**d**) Sample D, and (**e**) Sample E.

**Figure 11:**
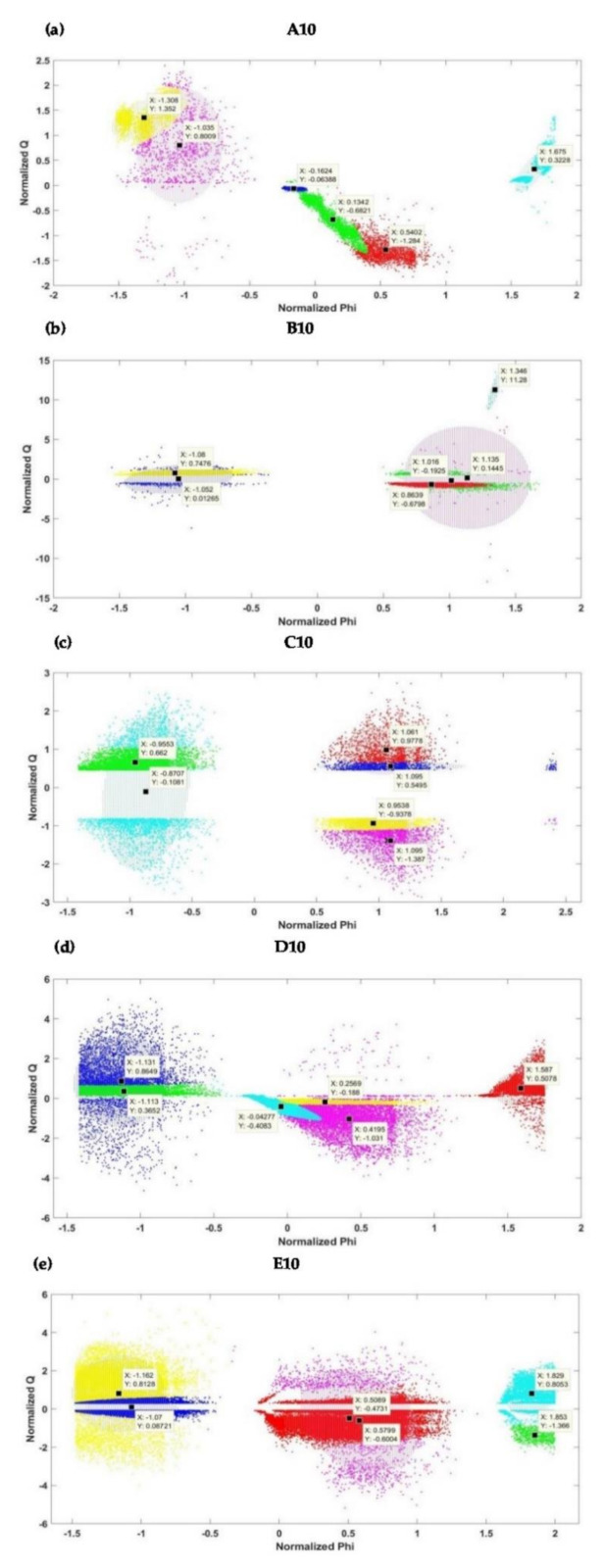
Clusters for the different samples at the final stage (interval 10): (**a**) Sample A (**b**) Sample B (**c**) Sample C (**d**) Sample D and (**e**) Sample E.

The six clusters with their centers and confidence ellipsoids as shown in Figure 10 indicate GMM models for the PRPD patterns of the initial stage (interval 1) of electrical treeing in Samples A, B, C, D and E. From Figure 10, it is clear that the ellipsoids are of different sizes and there are no restrictions to the orientation of their minor and major axis. Figure 11 shows the six clusters with their centers and confidence in the final stage (interval 10) of electrical treeing in Samples A, B, C, D and E ,which is close to the insulation breakdown. An interesting observation from Figure 11 is that none of the clusters are circular in shape and, therefore, K-means clustering could not be used here since it is not built to account for other shapes and a circular fit would be a bad fit to the data. In samples C and D, cluster overlapping can be clearly appreciated. The GMM algorithm produces the cluster centers based on the shape of the PRPD pattern and the PD activity and plots confidence ellipsoids with a 99% probability threshold as specified in the MATLAB program.

For each dataset sample, the normalized data is clustered into six groups, differentiated by color, using the GMM clustering. For each cluster in the two-dimensional (2D) plane, the midpoint of the cluster is also indicated in Figure 10 and Figure 11. In each case, the Phi and Q are normalized to return the vector-wise Z score of all the datasets (i.e., Samples A−E) with center 0 and standard deviation 1. The midpoints of the six clusters for each sample are shown in Appendix A.

For each dataset, the normalized data is clustered into six groups using the GMM clustering. From Figure 10 and Figure 11, it can be seen that the clusters are different, showing that the applied voltage and the stage of tree growth have a significant effect on the generated PD patterns. The initial and final stage clusters appear to be different for all the samples except for Sample A. For this sample, there is a similarity between the initial and the final stage clusters probably due to the continuous irregular trend in PD because of the formation of filamentary trees. The variance of the data in each cluster appears to be higher in the final tree stages when compared to the initial stages. This might be the PD mechanism over time and the increase in the number of PD events as the tree approaches the breakdown stage. It can be seen that the PRPD pattern clusters for all 5 samples at the initial stage appeared to be centered at different positions despite the fact that the applied voltages are close to each other. This shows that, even for the same applied voltage, the PD phenomenon in trees exhibits complex behavior and does not show any clear trend, making it difficult to evaluate in some instances. These conclusions can also be associated with the final stages of the treeing patterns in Figure 11.

As a further analysis of the GMM technique, Appendix A and Appendix B shows the cluster centers and their mean values/variances for all the samples (A−E), i.e., from the initial to the final stages of tree growth. In Appendix A, the cluster centers for the different sample datasets are shown. It can be seen that for samples A, B, and C, the positioning of the clusters from the beginning to the breakdown level varies significantly, although the first and the third cluster for all the samples A−E, fall within close range with insignificant variance in the *X*-axis in Appendix B. However, cluster 2 evidenced higher variance for samples A−C. This might be due to the stochastic nature of the PD mechanism of electric trees and the fact that the occurrence of the PD patterns within the first and last half of the AC power cycle are similar. Furthermore, it is interesting to note that the patterns for samples D and E have very similar pattern trends in the initial stage and the final breakdown stage, with insignificant variance among all the cluster shapes (see Appendix B, Table A6). This is a clear indication that with a lower breakdown time of insulation, treeing patterns are identical, and can easily be categorized. In the case of samples A−C, the results showed wider PD variability among the samples, showing their distinct nature. It was difficult to clearly differentiate between the initial and breakdown stage of the PD patterns. In addition, most of the pattern cluster centers show lower variance along the x-coordinates as compared to the y-coordinate, showing higher amplitude variation for the PD patterns as compared to the phase changes. The information in appendix B can serve as a statistical tool to predict and identify the applied voltages of the treeing patterns and their breakdown times for electrical trees in epoxy resin insulation. Assuming that there are new samples, they need to be classified as belonging to any of the confidence intervals of the samples in Appendix B and can be regarded as that particular sample.

In general, the results imply that the GMM can classify different degradation stages of the treeing patterns up to breakdown for samples having breakdown times higher than one hour, while it cannot effectively perform the same function for samples with shorter breakdown times, e.g., half an hour and lower. This might be because the PD mechanism has not been allowed to develop and it bridges the insulation in a shorter time, while more time before breakdown refers to a wider spread of treeing branches and more deterioration of the insulating material. To a certain extent, it can be said that the information in Appendix B almost correlates with the breakdown times but not necessarily the applied electrical stress across the sample defects. Although voltage can be regarded as one of the factors affecting the tree growth shape and structure in the cases analyzed here, the insulation breakdown time appears to be a key factor in determining the degradation levels of the PD patterns emanating from electrical trees.

### 5.3. Electrical Tree Pattern Recognition

**Figure 12:**
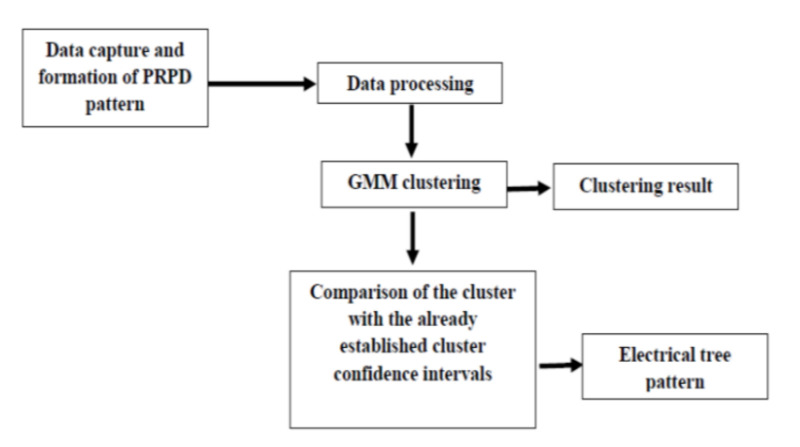
Flowchart for GMM Electrical tree pattern recognition.

As GMM is an easy and efficient technique for clustering, it has been shown, in the previous section, to aid the classification of electrical tree patterns. The fundamental flowchart for the electrical tree pattern recognition is shown in Figure 12. First, the data is captured and subsequent PRPD patterns are formed, followed by the data processing and the GMM clustering. The recognition of the tree growth clusters can be done by PD pattern judgment, i.e., comparing the clusters with the already established cluster confidence intervals in Appendix B. In this case, the level of the tree growth can be known, i.e., the initial, final, or other stages of degradation can be determined.

## 6. Conclusion

In this paper, the GMM has been utilized for clustering and classification applications in electrical trees emanating from epoxy resin insulation. Different PD samples were captured at different voltages from the initial to the final breakdown stage. The results show that PD dynamics vary with different stressing voltages and with the level of the tree growth. Depending on the sample and the applied electrical stress, there are different breakdown times. The GMM is chosen over other techniques in this work because it is robust and can perform hard clustering for complex data such as electrical tree patterns. The results clearly indicate that GMM can effectively classify patterns from the initial to the breakdown level for breakdown times above an hour, but not breakdown times of less than an hour, as the ones obtained with samples stressed at the highest stressing voltage of 16 kV. The PD patterns for shorter breakdown times possess identical clusters through the degradation stages. In this paper, the cluster centers and their confidence intervals have been developed to recognize the PD patterns in electrical trees at different stages ranging from the initial to the breakdown stage. However, the results presented in this paper can be further validated by experimenting with different samples captured at different voltages and breakdown times. Further research can also be conducted for different insulating materials such as polyethylene or cross-linked polyethylene to ascertain the efficiency of the proposed classification tool.

## Figures and Tables

**Table 1:** Samples, voltages, and the resulting time to breakdown of each experiment.

Sample	Voltage (kV)	Time BD (h)
A	12	3.8
B	14	1.5
C	14	1.4
D	16	0.6
E	16	0.47

**Table 2:** Statistical evaluation of Sample A at the first time interval.

	Count	Mean	Standard Deviation	Minimum	Maximum
Phi	10139.0	4.460 × 10^−1^	3.053 × 10^−1^	1.790 × 10^−5^	9.997 × 10^−1^
Q	10139.0	−1.297 × 10^−12^	4.454 × 10^−11^	−7.220 × 10^−11^	8.880 × 10^−11^
Phi (Stdscale)	10139.0	8.964 × 10^−17^	1.000 × 10	−1.460 × 10	1.813 × 10
Q (Stdscale)	10139.0	1.971 × 10^−18^	1.00 × 10	−1.591 × 10	2.022 × 10

**Table 3:** Akaike information criterion (AIC) values for dataset A at the first interval (A1).

*k* Value	Covariance Structure Σ			
	Diagonal-Shared	Full-Shared	Diagonal-Unshared	Full-Unshared
1	57,553	55,530	57,553	55,530
2	46,946	40,758	41,388	35,693
3	26,410	26,141	23,569	21,568
4	23,538	22,957	21,616	19,709
5	22,017	22,015	20,161	18,875
6	21,776	21,002	19,396	18,683
7	20,981	22,024	19,441	18,252
8	20,300	20,495	19,135	18,178
9	20,527	20,263	18,821	17,898
10	20,286	20,259	19,069	17,885
11	20,139	20,056	18,758	18,064
12	20,297	20,298	19,089	17,907

**Table 4:** Bayesian information criterion (BIC) values for dataset A at the first interval (A1).

*k* Value	Covariance Structure Σ			
	Diagonal-Shared	Full-Shared	Diagonal-Unshared	Full-Unshared
1	57,582	55,566	57,582	55,566
2	46,996	40,816	41,453	35,772
3	26,482	26,221	23,670	21,691
4	23,632	23,058	21,753	19,875
5	22,132	22,138	20,334	19,084
6	21,913	21,146	19,606	18,936
7	21,140	22,190	19,686	18,548
8	20,481	20,683	19,417	18,517
9	20,729	20,472	19,139	18,281
10	20,510	20,490	19,423	18,312
11	20,385	20,309	19,148	18,534
12	20,565	20,573	19,516	18,420

**Table 5:** The choice of best hyperparameter for dataset A.

Dataset	AIC		BIC	
	*k* Value	Σ Choice	*k* Value	Σ Choice
A1	4	Full-unshared	4	Full-unshared
A2	6	Full-unshared	6	Full-unshared
A3	5	Full-unshared	5	Full-unshared
A4	6	Full-unshared	6	Full-unshared
A5	8	Full-unshared	8	Full-unshared
A6	8	Full-unshared	8	Full-unshared
A7	7	Full-unshared	7	Diagonal-unshared
A8	5	Full-unshared	5	Full-unshared
A9	8	Diagonal-unshared	8	Diagonal-unshared
A10	6	Diagonal-unshared	6	Diagonal-unshared

**Table 6:** The choice of best hyperparameter for dataset B.

Dataset	AIC		BIC	
	*k* Value	Σ Choice	*k* Value	Σ Choice
B1	8	Diagonal-unshared	8	Diagonal-unshared
B2	6	Full-unshared	6	Full-unshared
B3	8	Full-unshared	8	Full-unshared
B4	6	Full-unshared	6	Full-unshared
B5	6	Diagonal-unshared	6	Diagonal-unshared
B6	6	Diagonal-unshared	6	Diagonal-unshared
B7	7	Diagonal-unshared	7	Diagonal-unshared
B8	8	Diagonal-unshared	8	Diagonal-unshared
B9	7	Full-unshared	6	Diagonal-unshared
B10	6	Full-unshared	6	Full-unshared

**Table 7:** The choice of best hyperparameter for dataset C.

Dataset	AIC		BIC	
	*k* Value	Σ Choice	*k* Value	Σ Choice
C1	4	Full-unshared	4	Full-unshared
C2	6	Full-unshared	6	Full-unshared
C3	6	Full-unshared	6	Full-unshared
C4	6	Full-unshared	6	Full-unshared
C5	5	Full-unshared	5	Full-unshared
C6	5	Full-unshared	5	Full-unshared
C7	6	Full-unshared	5	Diagonal-unshared
C8	6	Full-unshared	6	Full-unshared
C9	6	Full-unshared	5	Diagonal-unshared
C10	6	Full-unshared	6	Full-unshared

**Table 8:** The choice of best hyperparameter for dataset D.

Dataset	AIC		BIC	
	*k* Value	Σ Choice	*k* Value	Σ Choice
D1	5	Full-unshared	5	Full-unshared
D2	6	Diagonal-unshared	6	Diagonal-unshared
D3	6	Full-unshared	6	Full-unshared
D4	6	Full-unshared	6	Full-unshared
D5	6	Full-unshared	6	Full-unshared
D6	5	Full-unshared	5	Full-unshared
D7	6	Full-unshared	6	Full-unshared
D8	5	Full-unshared	5	Full-unshared
D9	6	Full-unshared	6	Full-unshared
D10	6	Full-unshared	6	Full-unshared

**Table 9:** The choice of best hyperparameter for dataset E.

Dataset	AIC		BIC	
	*k* Value	Σ Choice	*k* Value	Σ Choice
E1	6	Full-unshared	6	Full-Unshared
E2	6	Full-unshared	6	Full-Unshared
E3	6	Full-unshared	6	Full-Unshared
E4	6	Full-unshared	6	Full-Unshared
E5	8	Full-unshared	8	Full-Unshared
E6	6	Full-unshared	6	Full-Unshared
E7	7	Full-unshared	7	Full-unshared
E8	5	Full-unshared	5	Full-unshared
E9	6	Full-unshared	6	Full-Unshared
E10	5	Diagonal-unshared	5	Diagonal-unshared

**Table 10:** The parameter values for Gaussian mixture-based model (GMM).

Parameter	Value
Number of GMM components/clusters	6
Covariance matrix	Full
Shared covariance	False
Grid length	500
Number of iterations for the EM algorithm	1000

## Data Availability

Data available on request due to privacy.

## Appendix A

**Table A1:** Cluster centers for dataset A.

Sample	Cluster 1		Cluster 2		Cluster 3		Cluster 4		Cluster 5		Cluster 6	
	X	Y	X	Y	X	Y	X	Y	X	Y	X	Y
A1	−1.186	1.173	−1.125	0.387	−0.1354	−0.5698	0.1385	−0.8057	0.5116	−1.016	1.692	0.7549
A2	−1.056	−0.7532	−1.009	0.7146	−0.7347	−0.0112	−0.4768	−0.4107	0.8848	1.143	1.092	−0.251
A3	−1.058	−0.8859	−0.8825	−0.13	−0.7949	−1.403	0.8898	1.16	0.9781	0.03578	1.139	1.042
A4	−1.187	−0.0547	−1.157	0.7761	0.3293	−0.5711	0.7657	−0.8412	0.8846	0.2357	1.306	0.8736
A5	−1.006	1.686	−0.9573	0.2095	−0.5926	0.1435	0.859	−1.802	0.987	−0.2418	1.26	−0.3535
A6	−1.258	0.5283	−1.248	0.4826	−0.0548	0.8886	0.3141	−0.7269	0.5488	−1.603	1.241	0.5868
A7	−1.253	−0.3607	−0.4423	0.7335	0.07192	−0.3853	0.5229	−0.3716	0.5925	0.9693	1.06	−0.5146
A8	−1.461	1.946	−1.214	0.1791	−1.18	0.9087	0.4871	−0.8243	0.5937	−0.1102	1.912	0.854
A9	−1.256	0.2578	−1.057	1.301	−0.693	0.9227	0.5859	−0.897	0.9851	−1.136	1.791	0.1495
A10	−1.308	1.352	−1.035	0.8009	−0.1624	−0.0638	0.1342	−0.6821	0.5402	−1.284	1.675	0.3228

**Table A2:** Cluster centers for dataset B.

Sample	Cluster 1		Cluster 2		Cluster 3		Cluster 4		Cluster 5		Cluster 6	
	X	Y	X	Y	X	Y	X	Y	X	Y	X	Y
B1	−1.058	0.9892	−0.2775	0.0886	0.4043	−0.723	0.6774	−1.306	0.7123	−0.8706	1.966	0.7448
B2	−1.052	−0.897	−0.8772	−0.803	−0.7855	0.1771	0.8965	1.125	1.001	−0.1212	1.156	1.217
B3	−1.124	−0.871	−0.8612	−0.761	0.8251	0.7749	1.017	−0.0478	1.118	1.362	1.268	−0.0238
B4	−1.167	−0.6658	−0.9213	−1.19	−0.8323	−0.0097	−0.7085	−1.357	0.9122	1.061	1.134	0.4679
B5	−1.385	−1.039	−1.011	0.2924	−1.014	−0.2318	0.4921	0.7703	0.8353	−0.2362	1.903	−1.131
B6	−1.009	1.087	−0.8759	0.7558	−0.6402	0.2205	0.8734	−0.7698	1.076	−1.097	1.223	0.0073
B7	−1.064	0.7804	−0.942	0.9484	0.6927	−0.5958	0.9069	−2.673	1.033	−0.8764	1.079	0.3853
B8	−1.197	0.8737	−0.221	1.253	0.0151	−1.092	0.1591	−0.6668	0.425	0.3127	1.348	0.9801
B9	−1.185	0.7059	−1.107	0.2628	−0.1266	−0.3775	0.7336	−0.1164	0.8355	−0.2372	1.706	−0.1947
B10	−1.08	0.7476	−1.052	0.0127	0.8639	−0.6798	1.016	−0.1925	1.135	0.1445	1.346	11.28

**Table A3:** Cluster centers for dataset C.

Sample	Cluster 1		Cluster 2		Cluster 3		Cluster 4		Cluster 5		Cluster 6	
	X	Y	X	Y	X	Y	X	Y	X	Y	X	Y
C1	−1.059	0.8105	−1.05	0.7709	−0.9109	1.265	−0.4285	−0.5041	0.9417	−0.7893	1.037	−1.096
C2	−1.184	0.6434	−1.163	−0.7276	−0.9362	1.299	0.6173	−0.5202	0.9107	−1.187	1.396	0.4608
C3	−1.527	1.04	−1.228	1.053	0.1526	−0.5048	0.1862	−0.2235	0.2575	−1.061	1.653	0.638
C4	−1.32	0.8908	0.1122	−0.3929	0.1897	−0.3052	0.2012	−1.057	0.2261	−0.5983	1.631	0.6416
C5	−1.167	0.5778	−1.129	1.218	0.2461	−0.6028	0.4939	−1.508	0.5652	−0.4843	1.845	0.6457
C6	−1.057	0.5739	−0.9929	−1.147	−0.9911	0.7769	−0.8845	1.249	0.96	0.7948	0.965	−1.215
C7	−1.039	0.721	−1.035	−1.094	0.01662	1.051	0.879	−1.046	0.9277	0.7006	1.009	−1.649
C8	−0.9087	0.7963	−0.8923	−1.116	0.41	0.2699	0.9988	−1.02	1.072	−1.278	1.077	0.8119
C9	−0.9412	0.7026	−0.9178	−1.116	−0.8695	0.9657	1.041	0.701	1.055	−1.199	1.105	0.9908
C10	−0.9553	0.662	−0.8707	−0.1081	0.9538	−0.9378	1.061	0.9778	1.07	−1.384	1.095	0.5495

**Table A4:** Cluster centers for dataset D.

Sample	Cluster 1		Cluster 2		Cluster 3		Cluster 4		Cluster 5		Cluster 6	
	X	Y	X	Y	X	Y	X	Y	X	Y	X	Y
D1	−1.344	1.282	−1.246	0.4507	−0.1909	−0.3922	0.2312	−1.058	0.3376	−0.7218	1.624	0.7341
D2	−1.325	1.267	−1.269	0.3666	−0.2559	−0.2663	0.0334	−0.6915	0.4417	−0.9802	1.619	0.7413
D3	−1.582	1.118	−1.295	1.007	−0.2618	−0.213	−0.2295	−0.2193	0.2859	−0.8178	1.623	0.6534
D4	−1.262	0.8567	−0.448	−2.087	−0.2115	−0.0535	0.2842	−0.3403	0.3249	−0.962	1.648	0.7744
D5	−1.155	0.786	−1.13	0.33	−0.0899	−0.1165	0.4073	−0.8235	1.451	0.4504	1.69	0.6882
D6	−1.199	0.8595	−1.128	0.2565	−0.0391	−0.4619	−0.0344	−0.1949	0.4355	0.9097	1.64	0.4941
D7	−1.385	0.8288	−1.116	0.89	−0.1785	−0.1113	0.0824	−0.7388	0.4462	−1.074	1.571	0.5003
D8	−1.223	0.9173	−0.3391	−1.581	−0.1936	−0.097	0.2715	−1.239	0.3142	−0.5205	1.585	0.5408
D9	−1.042	0.9082	−0.9933	0.4894	−0.9854	0.2218	0.0139	−0.3329	0.4613	−1.136	1.638	0.5217
D10	−1.131	0.8649	−1.113	0.3652	−0.0428	−0.4083	0.2569	−0.188	0.4195	−1.031	1.587	0.5078

**Table A5:** Cluster centers for dataset E.

Sample	Cluster 1		Cluster 2		Cluster 3		Cluster 4		Cluster 5		Cluster 6	
	X	Y	X	Y	X	Y	X	Y	X	Y	X	Y
E1	−1.364	4.287	−1.25	0.1643	−1.048	0.4162	0.306	−0.9729	0.568	−0.1896	1.601	1.363
E2	−1.187	0.6647	−1.158	0.3211	−0.0214	−2.769	0.3147	−0.6605	0.9189	0.9922	1.642	0.7291
E3	−1.321	5.777	−1.04	0.2963	0.1226	−0.2736	0.282	−2.814	0.7842	−0.2625	2.089	1.537
E4	−1.102	0.4142	−0.2398	2.35	0.4716	−2.943	0.5297	−0.344	0.6166	−0.4342	1.94	0.4602
E5	−1.298	5.447	−1.154	0.0571	−1.13	2.824	0.4232	−0.0249	0.6845	−2.31	1.729	0.0095
E6	−1.155	2.081	−1.103	0.0718	0.4351	−1.365	0.4831	0.0177	1.784	0.06676	1.8	1.247
E7	−1.103	1.011	−1.053	0.3248	−0.9927	−0.0764	0.4207	−1.048	0.5579	−0.0067	1.773	0.6432
E8	−1.057	0.7747	−1.021	0.2494	0.5946	−0.3008	0.7169	−1.044	0.8952	−0.1777	1.984	0.3507
E9	−1.036	0.9421	−1.03	0.2727	0.5772	0.3024	0.5828	−0.1803	0.5843	−0.9298	1.873	0.3772
E10	−1.162	0.8128	−1.07	0.0872	0.5089	−0.4731	0.5709	−0.6004	1.829	0.8053	1.853	−1.366

## Appendix B

**Table A6:** Mean values of the cluster centers and their variances.

Sample		Cluster 1		Cluster 2		Cluster 3		Cluster 4		Cluster 5		Cluster 6	
		X	Y	X	Y	X	Y	X	Y	X	Y	X	Y
A	Mean	−1.2029	0.48886	−1.013	0.54543	−0.3947	−0.0141	0.422	−0.62	0.75064	−0.3	1.4168	0.34645
	Std.Dev	0.13659	1.01512	0.2303	0.40629	0.4713	0.7643	0.4153	0.737	0.20825	0.943	0.3172	0.564852
B	Mean	−1.1321	0.1711	−0.815	0.08594	−0.0598	−0.2537	0.6064	−0.523	0.90833	−0.06	1.4129	1.373295
	Std.Dev	0.10887	0.90572	0.3089	0.80068	0.7302	0.5543	0.5318	1.1006	0.21865	0.811	0.3254	3.54433
C	Mean	−1.1158	0.74183	−0.917	−0.266	−0.1739	0.3277	0.4165	−0.295	0.79859	−0.65	1.2813	0.07783
	Std.Dev	0.19142	0.14603	0.3803	0.95324	0.6944	0.851	0.6592	0.9567	0.32744	0.793	0.3219	0.984708
D	Mean	−1.2648	0.96884	−1.008	0.04874	−0.2449	−0.1898	0.1317	−0.583	0.49178	−0.59	1.6225	0.61561
	Std.Dev	0.15299	0.18377	0.3367	1.02863	0.2722	0.2047	0.191	0.3815	0.34311	0.701	0.0351	0.113455
E	Mean	−1.1785	2.22115	−1.012	0.41947	−0.0482	−0.4658	0.463	−0.767	0.92226	−0.24	1.8284	0.535085
	Std.Dev	0.11378	2.11348	0.2807	0.68629	0.7234	1.6552	0.1407	0.8244	0.48385	0.917	0.1518	0.830019
